# Prenatal Telepsychological Intervention for Preventing Anxiety: A Study Protocol

**DOI:** 10.3390/jcm13195877

**Published:** 2024-10-02

**Authors:** Alba Val, M. Carmen Míguez

**Affiliations:** Department of Clinical Psychology and Psychobiology, Faculty of Psychology, University of Santiago de Compostela, Campus Vida, 15782 Santiago de Compostela, Spain; alba.val@rai.usc.es

**Keywords:** pregnancy, prevention, perinatal anxiety, telepsychology

## Abstract

**Background:** Anxiety is one of the most frequent disorders during the perinatal stage that is associated with adverse health effects in women and their babies. In spite of this, preventive interventions during this stage are scarce. A long-distance intervention carried out during pregnancy can be an effective and accessible resource to help improve women’s emotional state. The objective of this study is to present and assess the effectiveness of a telepsychology cognitive–behavioral preventive intervention during pregnancy to manage anxiety. **Methods:** A random clinical trial will be carried out among pregnant women in Spain. The psychological intervention will take place via videoconference in seven weekly sessions, lasting one hour each, with groups of 6–8 pregnant women. Study outcomes will be collected via online questionnaires at five points in time: pre-intervention (baseline: t0), post-intervention (baseline: t1), follow-up at 1 month (t2), follow-up at 3 months (t3) and follow-up at 6 months (t4). The control group will receive usual pregnancy care (attendance at pregnancy follow-up consultations and information and answers to frequently asked questions provided by the midwife). Our primary hypothesis is that the intervention will decrease the frequency of women who present anxious symptomatology during pregnancy. The second objective is to analyze the effectiveness of this intervention to prevent depressive symptomatology during pregnancy, as well as postpartum anxiety and depressive symptomatology. The primary outcome measure is the difference in the mean anxiety score between the intervention and control groups assessed by the Edinburgh Depression Scale-Anxiety Subscale (EDS-3A), the State–Trait Anxiety Inventory (STAI) and Generalized Anxiety Disorder Screener (GAD-7) at the end of the intervention and at 1, 3 and 6 months postpartum. Generalized anxiety disorder (GAD) will be evaluated with the SCID clinical interview at the same time points. The secondary outcome will be determined by using the EPDS at the same time points. **Conclusions:** The results will determine whether a cognitive behavioral therapy applied via videoconference is well accepted by pregnant women, and if it is effective in preventing anxiety and emotional symptoms during the perinatal stage. If this intervention is an effective and useful resource among pregnant women, it can be implemented as a tool in Spanish healthcare.

## 1. Introduction

Anxiety is one of the most frequent disorders during the perinatal stage. Its prevalence is between 15% and 23% [1,2,3,4] during the pregnancy and between 3.7% and 15.0% [1,5] during postpartum. In Spain, the prevalence of anxiety ranges from 16.8 to 19.5% throughout pregnancy [3]. Additionally, one in ten women will experience comorbid anxiety and depression during pregnancy and one in twelve during the postpartum period [6]. The disparity in prevalence may be due to the moment of evaluation, the use of different evaluation instruments and cut-off points and cultural differences in relation to the importance given to mental health in a specific country and/or culture. Although its prevalence is high, research on treating anxiety during this period is recent [7,8].

Perinatal anxiety is associated with adverse health effects in women and their babies. Regarding mothers, it has been associated with an increased probability of developing postpartum depression [9], a higher risk of preeclampsia, obstetric complications and bonding problems [10,11,12]. As for newborns, they have been found to be more likely to have lower birth weight and poor cognitive development, among others [13,14], hence the importance of detecting and intervening early in prenatal anxiety to promote the well-being of mothers and children, because despite the negative consequences associated with perinatal anxiety, the majority of women are not identified or treated [7,15], as less than 15% receive treatment [16]. Given the high prevalence of prenatal anxiety and the potential for adverse consequences, early treatment is recommended [17,18]. Evidence on the effectiveness of psychological interventions during pregnancy is scarce [17,18,19,20], highlighting the importance of having specific interventions.

The need for greater access to psychological therapies has been highlighted by international institutions [17,20,21]. It must be taken into account that a non-pharmacological approach should be adopted in this period, due to the potential risks of using medications during pregnancy [22]. Furthermore, women prefer psychological interventions [23]. The National Institute for Health and Clinical Excellence [17] recommends psychological interventions, such as cognitive behavioral therapy, as a first-line treatment for mild and moderate prenatal anxiety. Early detection of anxiety symptoms in the perinatal stage can help prevent the development of more serious mental health problems [24].

As it is recommended for the prevention of depression during this stage [25], psychological interventions could be integrated into early childhood education classes, which would reduce the associated stigma and costs, and the benefits would extend to the baby before birth. During pregnancy, women frequently go to health services and are more willing to receive help because they believe it will have a positive impact on their baby [26]. However, there are barriers to accessing interventions during this period, such as lack of information about emotional problems, available and effective intervention options [27,28], as well as practical limitations of access to in-person treatment, such as costs, geographical distance, waiting lists or logistical problems such as attending appointments [26,27]. Furthermore, due to the stigmatization of mental health, especially during the perinatal period, women are reluctant to attend specialized mental health clinics in person [26,29].

The advances in information and communication technologies make it possible to have interventions via the internet, which can be carried out through computers, tablets or smartphones. These interventions may or may not be guided by a mental health professional, as well as being synchronous or asynchronous, that is, in real time or in deferred communication [30]. Furthermore, they have the advantage of helping to overcome the aforementioned barriers since they improve the acceptance of the intervention and are more flexible and accessible [30,31]. Systematic reviews of internet-based interventions during the perinatal period conclude that these interventions can reduce anxiety among mothers [32] and improve depressive and anxiety symptoms [31].

During the perinatal stage, interventions carried out through the internet represent a tool with great potential [33], as they tend to be more attractive since they reduce the need to travel—one of the barriers associated with low access to treatments for psychological problems during the perinatal period [30]. Peragallo-Urrutia et al. [34] found that 94.0% of pregnant and postpartum women use the internet, and 83% indicate their willingness to receive an online intervention in the perinatal period.

A form of therapy applied online is teletherapy, which has gained special importance since the COVID-19 pandemic. This form of treatment is carried out following a process similar to that of face-to-face therapy, although the interaction occurs electronically [35]. A systematic review of telepsychology [36] showed the effectiveness of different approaches, mostly based on the cognitive behavioral model, to reduce emotional distress. However, most of the research on the prevention of perinatal anxiety through the internet is unguided interventions, where participants access an application or web platform and view the material whenever they want and do not come in contact with an online healthcare professional at any moment, e.g., [37,38] or guided, where the participants, in addition to accessing the program material, have contact (via online and/or telephone) with a professional at some point during the intervention, e.g., [39,40]. To date, no protocol or study has been published on the effectiveness of cognitive behavioral therapy applied through video calls. Having an intervention developed through videoconferencing could help reduce the limitations of unguided therapies, since they do not offer direct interaction between the therapist and the patient, nor do they address the needs that may arise during said intervention [32].

On the other hand, it is worth remembering the high comorbidity of anxiety and depression. In a review and meta-analysis whose objective was to evaluate the effectiveness of psychological interventions to reduce perinatal anxiety, it was found that psychological interventions aimed at reducing anxiety during the perinatal stage were also effective in reducing symptoms of depression [7]. This supports research suggesting that transdiagnostic interventions targeting both anxiety and depressive symptoms tailored to the perinatal period may be more beneficial than disorder-specific interventions [37] and indicates the need to create transdiagnostic interventions to address symptoms of anxiety and depression in women during the perinatal stage since these comorbidities are generally not recognized or treated [41]. Therefore, considering the high frequency with which anxiety and depression occur together, treatments should treat them simultaneously [26].

To date, there is no protocol in Spain to prevent anxiety and comorbid emotional symptomatology during the perinatal stage by means of a videoconference psychological intervention. An intervention of this type could facilitate access and be helpful to many women during this period, reducing levels of anxiety and comorbid emotional symptoms.

The primary objective of the current study is to determine whether a preventive psychological intervention is superior to the usual care during pregnancy (attendance at pregnancy follow-up consultations and information and answers to frequently asked questions provided by the midwife) to prevent anxiety symptoms during pregnancy. The secondary objective is to examine whether this same intervention is more effective than usual care in preventing depressive symptoms during pregnancy, as well as anxiety and depressive symptoms postpartum.

## 2. Methods

### 2.1. Design and Participants

**Figure 1:**
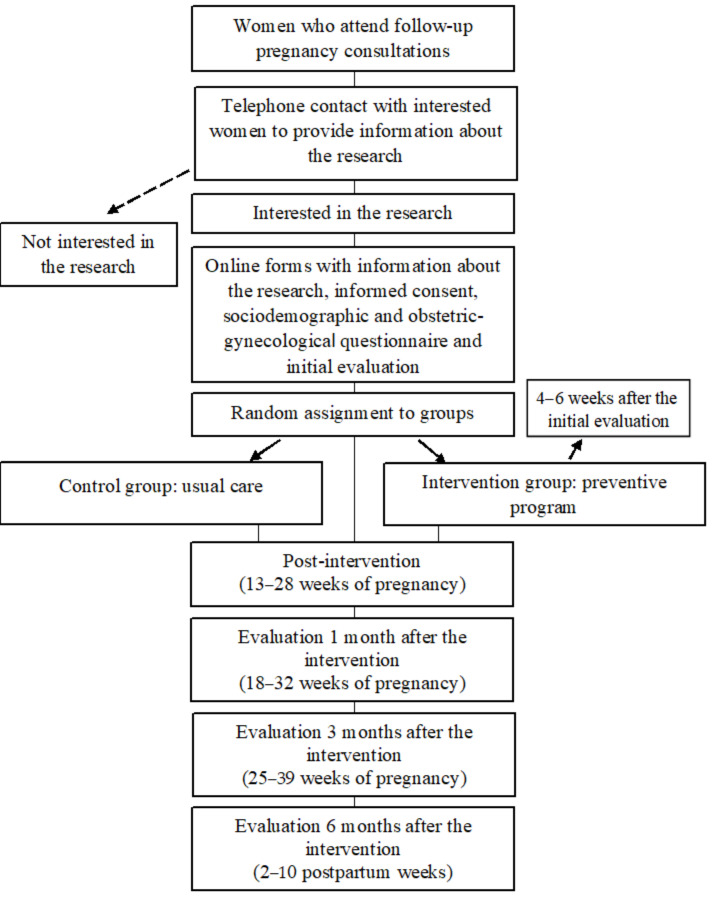
Flow chart of the study design.

The design (Figure 1) is a two-branch random controlled design with participants who will receive the intervention protocol assigned to the intervention group and those who will receive the usual care assigned to the control group. The intervention will begin between 4–6 weeks after the initial evaluation. Usual care consists of attending pregnancy follow-up and monitoring appointments, resolving any questions that may arise during the process with the midwives, and attending group prenatal preparation classes. Participants will be assessed before and after, and one, three and six months after finishing the intervention. The trial is registered on ClinicalTrials.gov (accessed on 20 September 2024). The registration ID is NCT06609291.

### 2.2. Recruitment of Women

The midwives who collaborate in the research will invite pregnant women who attend obstetrics and gynecology consultations for pregnancy control and monitoring and who will give birth to their children in public hospitals in the Servizo Galego de Saúde (Spain) to participate in the study. Subsequently, the personnel in charge of the research will contact the women by telephone and again inform them verbally and in writing about the objective and methodology of the study. Once the consent form has been signed, the different questionnaires will be sent electronically. A questionnaire regarding sociodemographic variables (e.g., age, marital status, education level, employment status and personal monthly income), obstetric history and current pregnancy (e.g., number of previous pregnancies, planned pregnancy, complications) will be developed for this study.

After completion of the baseline questionnaire, participants will be randomly assigned to either the intervention or control group using block randomization, in a 2:1 ratio, using an online randomization program. Study personnel are unaware of which treatment group each participant will be assigned to at the time of the baseline assessment.

### 2.3. Outcomes and Allocation of Participants to Trial Groups

#### 2.3.1. Primary Outcome

The primary outcome measure will be the difference in the mean anxiety scores between the intervention and control groups assessed by the EDS-3A, STAI and GAD-7 questionnaire before and at the end of the intervention, and one month, 3 months and 6 months after the intervention. Generalized anxiety disorder (GAD) will also be evaluated through the DSM-5 clinical interview at the same time points. In this case, the percentages found will be compared.

The Edinburgh Depression Scale-Anxiety Subscale (EDS-3A) is the anxiety subscale of the Edinburgh Postnatal Depression Scale (EPDS) [42]. Different studies, e.g., [43,44] have found that three items (3, 4, 5) can detect anxiety in women during the perinatal period. Each of the three items has four response options (0 = none, 1 = somewhat, 2 = quite a bit, and 3 = a lot). The total score of the subscale ranges from 0 to 9. The cut-off point used in the EDS-3A to detect anxious symptomatology will be ≥5 [45].

The State–Trait Anxiety Inventory (STAI) [46] assesses both the current level of anxiety and the individual’s predisposition to suffering from anxiety. It consists of 40 items, 20 of which refer to the state subscale (STAI-E), with the other 20 referring to the trait subscale (STAI-R). The score for each subscale ranges from 0 to 60, with higher scores indicating higher levels of anxiety. In this case, the State Anxiety Subscale (STAI-E) will be applied; the recommended cut-off point for women is greater than 31 (75th percentile).

Generalized Anxiety Disorder Screener (GAD-7) [47] is a self-administered instrument consisting of seven items with four response options. The total score ranges from 0 to 21, with the highest scores being the most severe. The GAD-7 has been validated for use in Spanish-speaking pregnant women [48]. The cut-off point used to detect anxiety symptoms will be ≥7 [49].

The SCID [50] is a semi-structured interview that determines formal diagnosis according to the Diagnostic and Statistical Manual of Mental Disorders (DSM-5). To diagnose generalized anxiety, you need to have had anxiety on most days in the last 6 months in at least three contexts of your daily life and show three of the six anxiety symptoms that are described in the manual. The use of such interviews improves diagnostic reliability by standardizing the assessment process and increases diagnostic validity by facilitating the application of DSM diagnostic criteria and the systematic enquiry for symptoms that might otherwise go unnoticed.

#### 2.3.2. Secondary Outcome

The secondary outcome measure will be the difference in the mean scores between groups on the EPDS at the same time periods described above. Depressive symptoms will be assessed using the EPDS [42], which is a self-reported questionnaire consisting of 10 items with four response options. The scores range between 0 and 30, with higher scores indicating greater severity of depression. The Spanish validation of the EPDS for use in pregnancy will be used [51]. The most appropriate cut-off point for screening for probable antenatal depression has been determined to be 10.

Additionally, acceptability and satisfaction with the program will also be evaluated through a satisfaction questionnaire.

### 2.4. Inclusion and Exclusion Criteria

Inclusion criteria include being pregnant with a gestational age ≤16, being over 18 years of age, understanding and speaking Spanish fluently, and giving consent to participate in the study. Additionally, participants must have access to the internet and a digital device (computer, tablet or smartphone). Those women who have a high-risk pregnancy or twins, are receiving pharmacological treatment for anxiety and/or depression or who do not give their consent will be excluded.

### 2.5. Adherence

Participants will be asked to participate in the activities proposed in each psychological content session and will be invited to put into practice what they learned during the week. To increase motivation and adherence, women will be able to contact the psychologist in charge of teaching the sessions if they have any questions or problems during the week.

### 2.6. Ethical Standards

This study is conducted according to the principles expressed in the Declaration of Helsinki, and has been approved by Comité Ético de Investigación de Galicia (CEIC) of Spain. All participants are guaranteed confidentiality of the information collected throughout the process. Participation will be completely voluntary and free, and no incentive (financial or otherwise) will be received for participation in the study.

### 2.7. Sample Size

Power calculations were conducted to determine the minimum sample size. As there was no research describing the efficacy of CBT delivered via videoconference in this population, power calculations were based on published RCTs of unguided CBT for antenatal depression [38]. To detect a between-group effect corresponding to Cohen’s d of 0.55, the minimum sample size for each group (α = 0.05, 80% power) was 53 participants. Therefore, we aimed to recruit a minimum sample of 140 participants to allow for expected attrition.

### 2.8. Analysis Plan

The primary analysis will compare the mean scores of EDS-3A, STAI-E and GAD-7 between the two groups before and after the intervention and one month, 3 months and 6 months after the end of the intervention to evaluate the effectiveness of the protocol in preventing anxiety symptoms. Similar analyses will be performed for the secondary result on depressive symptomatology. For the GAD, the frequencies in the two groups will be compared at the same evaluation periods.

Regarding the effectiveness of treatment, descriptive statistics will be calculated for all questionnaires. Variance analyses for repeated measurements will be performed to compare the differences between each initial score obtained with the different administered questionnaires and the scores at T1 (end of treatment), T2 (after 1 month), T3 (after 3 months) and T4 (after 6 months) will be evaluated. Bonferroni post hoc comparisons will also be conducted. The number of evaluations compared will need to be taken into account to adjust the *p*-value in the Bonferroni test with the aim of minimizing type 1 errors. The treatment effect will be calculated using Cohen’s d for each of the follow-up times. The Reliable Change Index will be calculated for each time in order to determine any significant clinical changes occurring in each individual case post-treatment. To compare differences in GAD, the chi-square test and Fisher’s exact test will be used in the contrast of 2 × 2 tables. In addition, Cochran’s Q test will be used to determine the existence of differences across time points and McNemar’s test will be used to compare the differences in categorical variables between time points in a paired manner.

Data will be analyzed using the Statistical Package for Social Science (SPSS) for Windows version 23.0.

## 3. Intervention

### 3.1. Rationale

The intervention was based on the principles of cognitive–behavioral therapy (CBT) and transdiagnostic treatments for emotional problems and includes three sections that address the main components of CBT: thoughts, pleasant activities and social contacts. CBT is one of the interventions that has obtained the best results for the treatment and prevention of perinatal anxiety [7,52,53]. On the other hand, transdiagnostic interventions may be more beneficial than specific interventions for each disorder, as scientific evidence suggests [7,37]. The need to develop this perinatal anxiety prevention program aimed at pregnant women is justified by the high prevalence of anxiety in the perinatal stage, as well as its possible consequences for both the mother and the baby [8,54]. Furthermore, there is a high comorbidity between symptoms of perinatal depression and anxiety [6], which indicates the importance of having a transdiagnostic intervention. Another aspect that highlights the need for a program of this type is the scarcity of existing psychological interventions to treat anxiety during pregnancy and postpartum [8,55]. A prevention program has been chosen because it would allow anxiety to be treated before it becomes a problem for the mother and her baby and would prevent future suffering [26]. Specifically, it would be a universal prevention program intended for all pregnant women regardless of whether or not they are at risk of anxiety. These programs have the advantage of increasing awareness about perinatal mental health as they provide information about its prevalence and risk factors, as well as tools to cope with emotional distress and increase the support network.

A CBT prevention program has been selected for development because it is found to obtain some of the best results in clinical practice [7,24]. The techniques included in the program, such as cognitive restructuring, behavioral activation or social support, have been selected because they repeatedly appear in the reviewed interventions that have shown greater effectiveness [7,8,24,32]. It should be noted that the intervention will not exclusively address anxiety, but also depression, due to the high comorbidity they present [6].

Regarding the method of application, it will be carried out at a group level, because group interventions obtain good results and involve lower costs, high satisfaction for users and high rates of compliance with the program [24]. Likewise, an electronically developed intervention has been chosen due to the benefits that this type of modality provides, such as lower cost and more convenience [31]. To ensure a safe and comfortable environment for the group participants, in the first session, some basic rules will be established for the proper functioning of the group. The therapy will be carried out by a psychologist via videoconference, which will reduce the disadvantages of online interventions, such as there being no interaction between the therapist and the patient or not attending to the needs that may arise during it [32].

To date, in Spain, we do not have any specific intervention protocol to address anxiety in pregnancy. We consider that it could help reduce its prevalence and the possible consequences it has on mothers and children, as well as promote the psychological well-being of women during pregnancy and postpartum. At the same time, being a transdiagnostic intervention, it would also allow comorbid depression symptoms to be treated.

### 3.2. Overview of the Intervention

The psychological intervention will consist of seven group sessions, conducted weekly, with a duration of approximately 60 min each, with the exception of the first and last ones, which will last an hour and a half since they will involve carrying out the presentation and evaluation of the intervention, respectively. In turn, the program will be divided into three modules. Module 1: psychoeducation and awareness about perinatal mental health, which provides information on the prevalence of anxiety symptoms in the perinatal stage and the consequences for mothers and babies. Module 2: Management of emotions and thoughts. It covers three sessions and aims to provide strategies to identify and restructure those thoughts that harm us, as well as recognize the behaviors and emotions that they generate. Module 3: aimed at teaching how to solve problems effectively and communicate in the best possible way.

Throughout the sessions, relaxation training will be carried out in order to have a technique for reducing anxiety when it appears. Likewise, in all sessions, exercises will be assigned to do at home so that the participants can practice what they have learned each week. Finally, a closing session will be held in which the skills learned throughout the intervention will be reviewed. Each session will have a similar structure: review of homework assignments and resolution of problems that have arisen in this regard, explanation of the content of the session, practical exercises, homework assignment and relaxation training. The components of the intervention are described below and in Table 1.

Module 1: Psychoeducation and Awareness about Perinatal Mental Health

*Session 1: Learning about this new stage.* Welcome and a general presentation of the program and psychoeducation on the prevalence of emotional symptoms in the perinatal stage and its consequences for mothers and children. The role of hormones during motherhood will also be explained, both for the correct development of the pregnancy and its possible effects on physical and emotional levels. The activities will consist of debates and the normalization of emotions. The session will end with the practice of guided relaxation. As homework, an audio will be sent to practice relaxation.

Module 2: Management of Emotions and Thoughts

*Session 2: Becoming aware of my emotions*. Psychoeducation on the changes and demystification of the myths associated with motherhood. Information will be provided about the usefulness of emotions and their components. The changes and myths associated with motherhood will be discussed; the goal is to learn to identify and validate both positive and negative emotions. As homework, they will be given a self-reflection exercise to learn to identify the components of an emotion (physical, cognitive and behavioral parts), with the aim that they learn to detect emotions before they become uncontrollable. Additionally, an audio will be sent to practice guided relaxation.

*Session 3: Becoming aware of my thoughts I*. How they affect my emotions. Information will be provided about the formation of our thoughts and their relationship with our emotions. A metaphor about the functioning of negative thoughts will be read and discussed. The practical exercises will consist of interpreting a daily life situation and seeing how this interpretation affects your thoughts. As homework, they will self-record their thoughts and the emotions they have generated. Likewise, they will practice a new relaxation technique, diaphragmatic breathing.

*Session 4: Becoming aware of my thoughts II*. How they affect my behavior. Psychoeducation on thoughts will continue and cognitive distortions associated with this stage will be discussed. Some vignettes will be presented to identify how thoughts influence our emotions and behaviors. Techniques will be provided to generate alternative thoughts. Likewise, the functioning of behavioral activation will be explained. The practical part will consist of discussing the vignettes and generating alternative thoughts. On the other hand, they will be asked about pleasant activities to launch behavioral activation. As homework, they will generate alternative thoughts and record the emotions that accompany them. They will also perform diaphragmatic breathing and begin behavioral activation.

Module 3: Training in Problem-Solving and Assertiveness Skills

*Session 5: Deciding calmly.* Psychoeducation on how problems influence our mood and different coping strategies. Through practical exercises, it is intended that each woman identifies her way of making decisions and learning new strategies. The session will end by introducing a new relaxation technique, progressive muscle relaxation. As homework, alternatives will be generated for a daily problem raised during the session, behavioral activation will be maintained and, with the help of an audio recording, they will practice the new relaxation technique.

*Session 6: Learning to communicate better.* Information will be provided about the different forms of communication and their consequences. Through everyday situations, it is intended that each woman identify her way of communicating. Assertive communication strategies will be practiced. Homework will include practicing assertiveness in everyday life situations and practicing progressive muscle relaxation and behavioral activation.

*Session 7: More tools for this new stage.* The skills learned during the sessions will be reviewed. The expectant mothers will then be presented with a list of community support resources. Their interest and participation will be appreciated, and the group will end by carrying out the relaxation practice that they have chosen. Finally, the post-treatment evaluation will be carried out and the day for the first follow-up will be agreed upon.

## 4. Discussion

Perinatal anxiety is very common and can have important consequences for women, their children and their families [52]. To avoid these negative consequences, healthcare institutions and providers must emphasize the need to implement preventive interventions. Although pregnancy is a good time to carry out such interventions [56], during this period, help-seeking rates are low [16], mainly because most women are not aware of having a psychological problem and attribute their symptoms to pregnancy or childbirth. Barriers such as lack of information, the cost of conventional treatments or possible stigmatization also contribute to this [57,58].

Online interventions, including telepsychological interventions, offer advantages in overcoming the barriers mentioned above, facilitating access to reduced-cost interventions and offering greater flexibility [31]. Furthermore, these types of interventions make it possible to reach women who have more difficulty traveling to receive assistance [59] or have logistical problems attending in-person appointments [26,27], which allows adherence to be increased [39]. Likewise, group interventions have been shown to be effective in reducing emotional symptoms during this stage and show a high rate of compliance, acceptance and satisfaction on the part of women [8], in addition to reducing costs compared to an individual intervention [24].

However, telepsychological interventions also have limitations. Llerena and Ramos-Nobo [46] have classified them into five blocks: technical, ethical, sender, receiver and practical aspects. Considering the technical aspects, Maheu et al. [60] point out the need to have access to the internet and know how the devices used work, something that may not be available to everyone. Regarding ethical aspects, problems related to the privacy and/or confidentiality of information stand out [61,62]. Regarding the limitations associated with the sender, that is, the therapist, some resist integrating technological resources into professional practice [63] and/or do not have the competence (skills) to develop an intervention electronically [62]. Regarding the inconveniences related to the receiver, it should be noted that not all patients know how to use technological resources, nor are comfortable in the absence of face-to-face interactions [64]. Finally, on a practical level, the difficulty in establishing the alliance between patients and therapists and the difficulty in transmitting empathy stands out due to the absence of eye contact [65].

The therapies developed through videoconferencing make up for certain disadvantages of a purely online therapy, in which there is no direct contact with a professional, such as not offering direct interaction between the therapist and the patient, or attending to the needs that may arise during the same [32]. Therefore, whenever an online therapy is developed, it is important to consider the participation of a professional during the intervention and adapt it to the needs of women [66].

Likewise, knowing the high rate of comorbidity between anxiety and depression during the perinatal stage [6], a transdiagnostic intervention could be more beneficial than specific interventions for each disorder [37], as it allows addressing both symptoms simultaneously.

The therapies carried out over the internet do not replace face-to-face therapies developed in the clinical setting, although this modality can be a cost-effective, easily accessible and low-intensity resource for women who cannot receive standard psychological treatment.

This intervention is developed with the aim of contributing to increasing scientific evidence on the effectiveness of CBT applied by videoconference to prevent emotional symptoms during pregnancy and postpartum. This protocol aims to promote the prevention of anxiety and emotional symptoms associated with the perinatal stage. At the public health level, it is necessary to address maternal mental health and dedicate resources to provide tools to future mothers to cope with anxiety symptoms during the perinatal stage, which may reduce the possible adverse consequences that may arise from them.

## Figures and Tables

**Table 1:** Intervention components.

Module 1. Psychoeducation and Awareness about Maternal Mental Health			
Session 1. Learning about This New Stage			
Welcome	General Program Presentation		Psychoeducation
			-Information about emotional symptoms and their consequences during the perinatal stage -Hormonal changes -Relaxation
**Module 2. Management of Emotions and Thoughts**			
**Session 2**	**Session 3**		**Session 4**
**Becoming aware of my emotions**	**Becoming aware of my thoughts I**		**Becoming aware of my thoughts II**
-Information about the myths and changes associated with motherhood -Identify and validate your emotions -Relaxation	-Psychoeducation on the formation of our thoughts and their influence on our emotions -Identify your thoughts -Relaxation		-Psychoeducation on how thoughts affect our behavior and behavioral activation -Identify your cognitive distortions and learn strategies to generate alternative thoughts -Identify pleasant activities and begin behavioral activation -Relaxation
**Module 3. Training in Problem-Solving and Assertiveness Skills**			
**Session 5**		**Session 6**	
**Deciding calmly**		**Learning to communicate better**	
-Coping and problem-solving strategies -Behavioral activation -Relaxation		-Information about social support, assertive communication guidelines and seeking support -Behavioral activation -Relaxation	
**Session 7. More tools for this new stage**			
-Farewell and acknowledgements -Information about existing support resources -Relaxation			

## Data Availability

The datasets generated and/or analyzed during the current study will be available from the authors on reasonable request.
